# Effect of boron toxicity on pollen tube cell wall architecture and the relationship of cell wall components of *Castanea mollissima* Blume

**DOI:** 10.3389/fpls.2022.946781

**Published:** 2022-07-26

**Authors:** Weiwei Zhang, Qing Zhang, Yu Xing, Qingqin Cao, Ling Qin, Kefeng Fang

**Affiliations:** ^1^Beijing Advanced Innovation Center for Tree Breeding by Molecular Design, Beijing University of Agriculture, Beijing, China; ^2^Key Laboratory for Agricultural Application and New Technique, College of Plant Science and Technology, Beijing University of Agriculture, Beijing, China; ^3^Beijing Bei Nong Enterprise Management Co. Ltd, Beijing, China; ^4^College of Landscape Architecture, Beijing University of Agriculture, Beijing, China

**Keywords:** *Castanea mollissima* Blume, boron toxicity, pollen tube, pectin, callose, ultrastructure

## Abstract

Boron (B) is essential to plant development. However, excessive B is toxic to plants. This research was performed to evaluate the effects of B toxicity on cell wall architecture of Chinese chestnut (*Castanea mollissima* Blume) pollen tubes with emphasis on the relationship among pectins, cellulose, and callose. Results showed that 0.8 mM H_3_BO_3_ inhibited pollen germination and led to abnormal morphology of the pollen tubes. B toxicity also affected the distribution of cell wall components of the pollen tube. In control pollen tube, esterified and acid pectins were distributed unevenly, with the former mainly at the tip and the latter on the distal region. Cellulose was distributed uniformly on the surface with less at the tip; callose reduced gradually from base to sub-tip of the pollen tubes and no callose at the tip of the tube was detected. B toxicity led to the deposition of esterified and acid pectins, cellulose, and callose at the tip of the pollen tube. Results from scanning electron microscopy and transmission electron microscopy showed that B toxicity also altered pollen tube wall ultrastructure. The results from enzymatic treatment illustrated that there existed a close relationship among pectins, cellulose, and callose. B toxicity also altered the relationship. In a word, B toxicity altered deposition and relationship of pectins, cellulose, and callose of pollen tube wall.

## Introduction

Boron (B) is essential to the normal development of higher plants (Warington, 1923; Brdar-Jokanović, 2020). To date, B is defined as a crosslinking molecule in plant cell walls (Landi et al., 2019) and plays a structural role in the plasma membrane (Voxeur and Fry, 2014). The concentration range is narrow from deficiency to toxicity for B in higher plants (Matthes et al., 2020; Behera et al., 2022). B toxicity influences various developmental processes such as hindering glutathione (Ruiz et al., 2003) and tocopherol formation (Keles et al., 2004), decreasing root cell division (Aquea et al., 2012), inducing reactive oxygen species (ROS) (Cervilla et al., 2007), changing the photosynthesis (Landi et al., 2013; Papadakis et al., 2018), expression of genes (Sakamoto et al., 2011; Aquea et al., 2012) altering signal transduction and biomacromolecule metabolism, thickening cell wall irregularly (Huang et al., 2014; Sang et al., 2017; Seth and Aery, 2017; Papadakis et al., 2018; Wu et al., 2018), and losing the balance of the C/N ratio (Sotiras et al., 2019). The research on leaf tissue of two citrus rootstocks showed that B toxicity resulted in damage to the pectin crosslinking structure, B deficiency induced more accumulation of cellulose (Wu et al., 2018), and miRNA397a regulated secondary cell-wall biosynthesis to tolerate the long-term B toxicity by targeting two laccase genes, *LAC17* and *LAC4* (Huang et al., 2016).

In angiosperm, the pollen tube carries a pair of sperm cells to fertilize the egg cell and the central cell, respectively, in the embryo sac to complete double fertilization. During this process, pollen tubes grow from their tips very fast and demand a great quantity of membrane and cell wall precursors to form the new cell wall (Zhang et al., 2010; Bou and Geitmann, 2011). Geitmann and Steer (2006) summarized that the pollen tube cell wall had several functions in growth including acting as physical control of cell shape, protecting the generative cell (sperm cells) from mechanical damage, resisting the turgor pressure, and adhering to the transmitting tissue. Thus, the pollen tube cell wall takes an active part in the process of fertilization. The pollen tube wall has been reported to be consisting of cellulose, callose pectins, and other components, such as hemicelluloses and proteins (Li et al., 1996, 2002; Ferguson et al., 1998). Esterified pectins are produced by the Golgi apparatus and transported to the pollen tube extreme tip (Hasegawa et al., 1998), where the esterified pectins are de-esterified to be acid pectins by the enzyme pectin methyl-esterase (PME) (Li et al., 2002). The crosslinks between the acidic residue of acid pectins and Ca^2+^ ions form a semi-rigid pectate gel (Braccini and Perez, 2001), which supports mechanically pollen tube elongation.

As a monoecious plant,the ratio of female and male flowers can reach 1:2400–4000 in Chinese chestnut (*Castanea mollissima* Blume). The male flowers consumed much nutrition, which influenced nutrition distribution and development of female flowers, and even causing empty bur phenomenon. And in field production, farmers sprayed boric acid to increase the fruit setting percentage. However, the amount of boric acid sprayed was difficult to measure, and the long-term spraying would induce boron accumulation in plants. Therefore, the study on the effects of B toxicity on Chinese chestnut pollen germination and pollen tube wall components is of great significance to improve yield.

As we know, B is essential for pollen germination and tube growth (Obermeyer et al., 1996; Fang et al., 2016), making the pollen a good system with which to study the effect of B toxicity. In our previous research, we found that B toxicity inhibited apple pollen tube growth by disrupting Ca^2+^ influx (Fang et al., 2016). However, the detailed effects of B toxicity on pollen tube wall components and ultrastructure remain to be clarified. In the present study, *Castanea mollissima* pollen was used as the material to study the influence of B toxicity on pollen tube growth, with focus on cell wall components, ultrastructure, and relationship among pectins, cellulose, and callose.

## Materials and methods

### Plant materials and culture procedure

Mature pollen grains were collected on 10 June 2018 from *C*. *mollissima* in the Chestnut Experiment Station in the Huairou District of Beijing, China. Then, they were placed on paper towels for 15 h at room temperature, and were stored in a refrigerator at -20°C.

The culture medium for *C*. *mollissima* pollen grain included 15% (w/v) sucrose and 3.6 mM CaCl_2_. Different concentrations of H_3_BO_3_ (Sigma, St. Louis, MO, USA), 0, 0.08 mM, 0.4 mM, and 0.8 mM, were added to the culture medium at the beginning, pH 5.8. The pollen grains were cultured at 34°C in the darkness.

After 27 h of culture, the pollen germination rates were calculated as previously described (Fang et al., 2016) under a BX51 microscope (Olympus, Japan) with a CoolSNAP HQ CCD camera (Photometrics). Five hundred pollen grains were counted in each experiment to calculate germination rates, which was repeated three times. The length and diameter of 150 pollen tubes were measured with MetaMorph (Universal Imaging). Three independent experiments were performed. Based on the germination rate and morphology of the pollen tube (the swelling of the tube tip), the concentrations of boron at 0.08 mM and 0.8 mM were used as control and toxicity treatment for further tests, respectively.

### Enzymatic treatment

After being cultured for 27 h, the pollen tubes were treated with 1% cellulase, pectinase, and β-1,3-glucanase (soluble in 100 mM Phosphate buffer, PBS) (Sigma, St. Louis, MO, USA), respectively, to detect the relationship among pectins, cellulose, and callose.

### Fluorescent localization of cell wall components

The cultured pollen tubes were fixed with 3% paraformaldehyde (soluble in 100 mM PBS) before labeling and staining. The esterified and acid pectins of pollen tubes were reacted first with the monoclonal antibodies JIM7 and JIM5 (provided by Dr Knox JP, diluted at 1:20), then with the secondary antibody FITC-labeled sheep anti-rat IgG (Sigma, St. Louis, MO, USA, diluted at 1:100 in PBS) according to Fang et al. (2016). The labeled pollen tubes were observed under the laser scanning confocal microscope (LSCM, Leica Co., Germany) with the excitation wavelength at 488 nm and emission wavelength at 515 nm, respectively. The above procedure without the monoclonal antibody was set as control.

For cellulose localization, the cultured pollen tubes were incubated with primary probe CBM3a (diluted at 1:100 in PBS) for 1 h, and second antibody:mouse anti-HIS (diluted at 1:100 in PBS), and finally incubated with tertiary antibody:anti-mouse:FITC (diluted at 1:50 in PBS). For callose localization, pollen tubes were first incubated with the monoclonal antibody to (1 → 3)-β-glucan (Cat. No. 400-2, Biosupplies Australia, diluted at 1:50 in PBS) and then incubated with the second antibody:anti-mouse:FITC (diluted at 1:50 in PBS). The labeled pollen tubes were observed under the LSCM (Leica Co., Germany) with the excitation wavelength at 488 nm and emission wavelength at 515 nm, respectively.

The relative intensity of the fluorescent signals from esterified and acid pectins, cellulose, and callose was measured with Image-Pro Plus 6.0 software (http://www.mediacy.com/) according to Fang et al. (2016). At least six pollen tubes of each treatment were measured. The fluorescent signals were measured along with the tube wall from the pole of the tube to the pollen grain, and the average fluorescence intensity was shown; the data of intensity were shown in Supplementary Table S1.

### Fourier transform infrared analysis of tube wall components

The cultured pollen tubes were collected by centrifugation, then washed three times with deionized water, and dried in a layer on a barium fluoride wafer in 28°C incubators. The infrared (IR) spectra of the pollen tubes were recorded using a MAGNA 750 FTIR spectrometer (Nicolet Corp., Japan). At least nine pollen tubes were tested for each treatment, which was repeated three times.

### Scanning electron microscopy (SEM)

For scanning electron microscopy, 2.5% glutaraldehyde in 100 mM PBS (pH 7.2) with 2% (w/v) sucrose was applied to fix pollen tubes for 2 h. Next, the pollen tubes were washed with 100 mM phosphate buffer. After that, the pollen tubes were post-fixed in 2% osmium tetroxide (soluble in deionized water) for 1 h, dehydrated in an ethyl-alcohol series, and then displaced with tret-butanol, and finally dried with a VFD-215 freeze-drying apparatus. Dried pollen tubes were placed on SEM stubs, coated with gold-palladium (Polaron SC7620; VG Microtech, Uckfield, UK), and examined with a TESCAN 5136 SEM (TESCAN Com., Czech).

### Transmission electron microscope (TEM)

For transmission electron microscopy, 2.5% glutaraldehyde in 100 mM PBS (pH 7.2) with 2% (w/v) sucrose was applied to fix pollen tubes for 2 h. After that, the pollen tubes were washed with 100 mM phosphate buffer. Then, the pollen tubes were post-fixed in 2% osmium tetroxide (soluble in deionized water) for 1 h, dehydrated in an ethyl-alcohol series, and finally embedded in Spur resin. Ultrathin sections (50 nm) were cut on an ultramicrotome (EM UC6&FC6, Leica, Germany), examined with a TEM (JEOL Ltd., Tokyo, Japan), and imaged with a CCD camera (H7650, HITACHI, Japan).

### Statistical analysis

Sigma Plot 10.0 was used to produce line charts of the data. IBM SPSS Statistics v20 was used for statistical analysis, and data were analyzed by ANOVA and Duncan's test. All statistical tests were performed at *P* = 0.05.

## Results

### Effects of B toxicity on pollen germination and tube growth

B affected pollen germination. Pollen germination rate was 21.89% without H_3_BO_3_, while it was 31.60% with 0.08 mM H_3_BO_3_. However, 0.8 mM H_3_BO_3_ made the germination rate decrease to 14.57%. In the following research, 0.08 mM H_3_BO_3_ was used as control, while 0.8 mM H_3_BO_3_ was used as a B toxicity treatment.

B also affected pollen tube growth. After 27 h of culture, the tube length was only 15.08 μm without H_3_BO_3_, while it was 38.22 μm and 39.82 μm with 0.08 mM and 0.8 mM H_3_BO_3_, respectively (Table 1). No obvious difference existed concerning the pollen tube length of control and B toxicity.

**Table 1:** Germination rate and length of *C*. *mollissima* pollen tubes under different concentrations of H_**3**_BO_**3**_.

**H**_3_ **BO**_3_	**Germination**	**Pollen tube**	**Pollen tube**
**treatment/mM**	**rate/%**	**length/**μ**m**	**width/**μ**m**
0	21.89 ± 1.68 ab	25.80 ± 0.75 b	5.53 ± 0.26 c
0.08	31.60 ± 0.45 a	38.22 ± 0.36 a	5.71 ± 0.20 c
0.4	19.78 ± 1.55 b	39.10 ± 0.53 a	16.23 ± 3.34 b
0.8	14.57 ± 1.04 c	39.82 ± 2.84 a	20.91 ± 1.37 a

*Different letters indicate statistically significant differences between pollen tubes grown in various condition (P ≤ 0.05)*.

**Figure 1:**
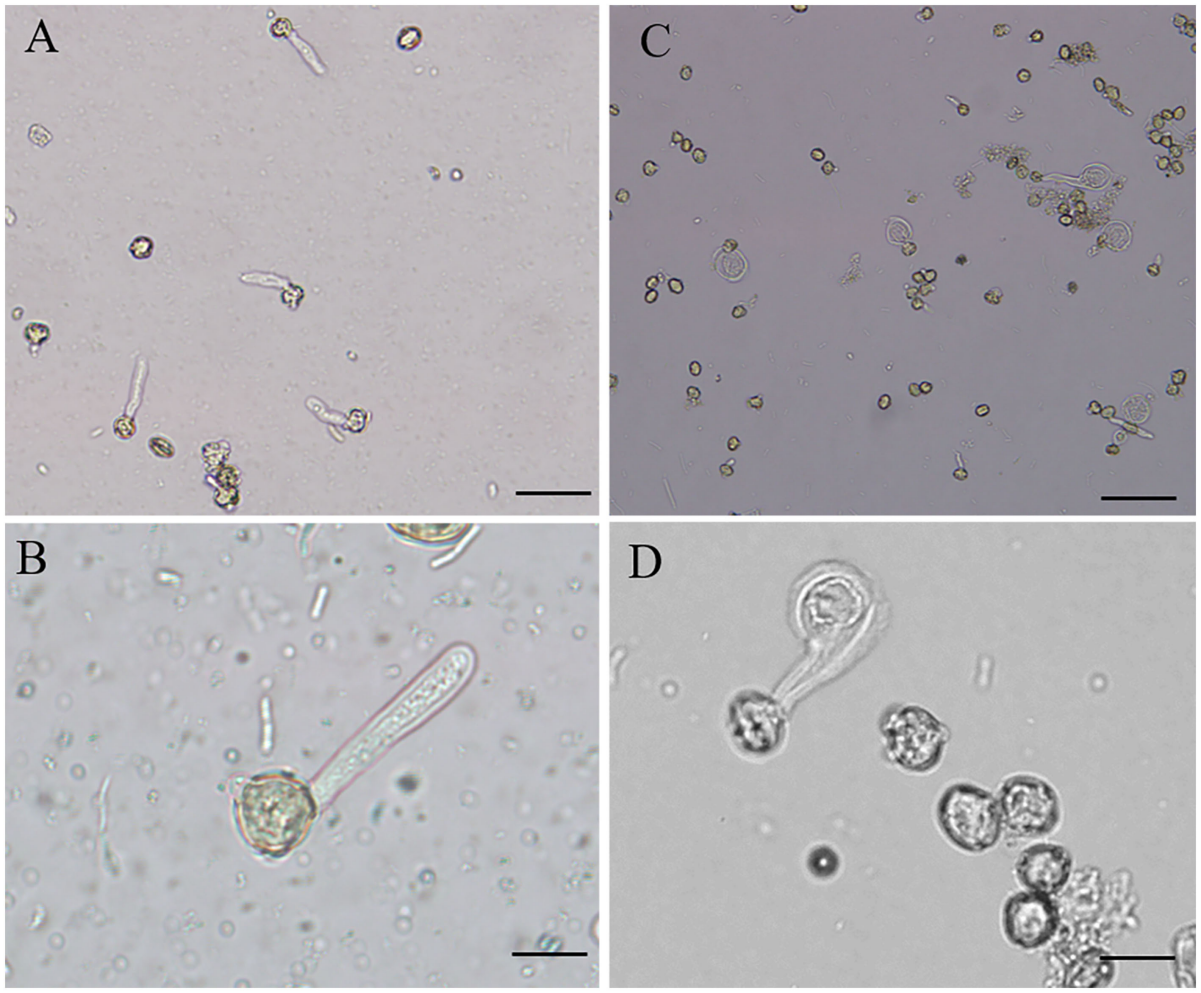
Morphology of *Castanea mollissima* pollen tubes under different culture conditions. Bar = 50 μm in A and C. Bar = 20 μm in B and D. **(A,B)** Pollen tubes in control medium. **(C,D)** Pollen tubes in B toxicity medium.

B toxicity led to abnormal morphology of pollen tube. Control pollen tubes were straight with a slender uniform diameter (Figures 1A,B); however, B toxicity-treated pollen tube tip was swollen with a larger diameter (Figures 1C,D). The tube width is shown in Table 1, and the tube width was 5.53 μm without H_3_BO_3_ and 5.71 μm with 0.08 mM H_3_BO_3_, while it reached 20.91 μm with 0.8 mM H_3_BO_3_.

### Effects of B toxicity on pectins of pollen tube

**Figure 2:**
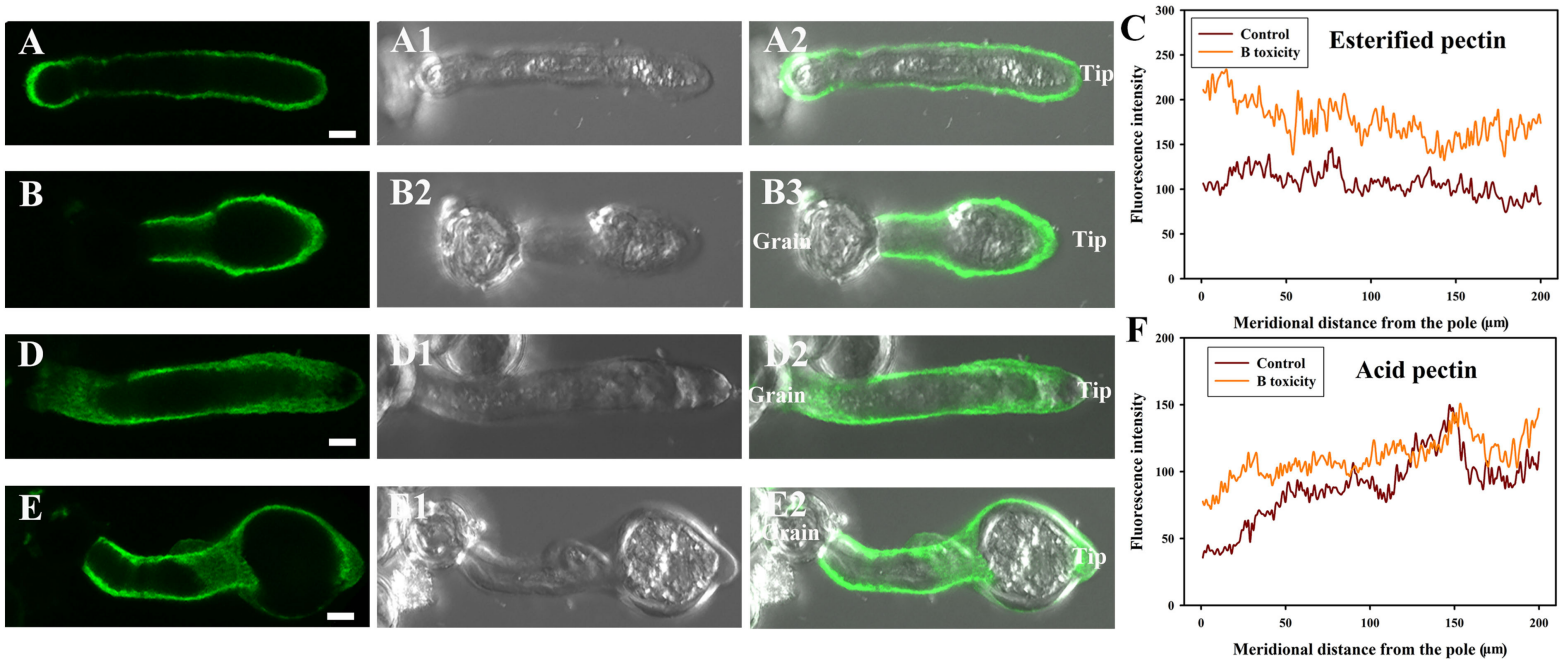
Influence of B toxicity on esterified and acid pectins distribution of *C*. *mollissima* pollen tube wall. **(A)** Esterified pectins in the control pollen tube indicated by JIM 7 labeling. **(B)** Esterified pectins in B toxicity-treated pollen tube. **(C)** Quantitative analysis of the fluorescent intensity of esterified pectins in the wall of control (Control) and B toxicity-treated pollen tubes (0.8 mM H_3_BO_3_). **(D)** Acid pectins in control pollen tube indicated by JIM 5 labeling. **(E)** Acid pectins in B toxicity-treated pollen tube. **(F)** Quantitative analysis of acid pectins fluorescent intensity in the wall of control (Control) and B toxicity-treated pollen tubes. Bar = 5 μm.

B toxicity induced more esterified pectins at the pollen tube wall (Figure 2). In the control pollen tube, esterified pectins distributed uniformly along the whole tube (Figure 2A), while B toxicity induced much more esterified pectins along the whole tube (Figure 2B). Fluorescent intensity data supported the above results (Figure 2C).

B toxicity affected the distribution of acid pectins on pollen tubes. In the control pollen tube, acid pectins are mainly distributed on the surface beyond the tip (Figure 2D). B toxicity strengthened acid pectins at the wall near grain and tip (Figure 2E). Fluorescent intensity supported the above results (Figure 2F).

### Effects of B toxicity on cellulose and callose of pollen tube

**Figure 3:**
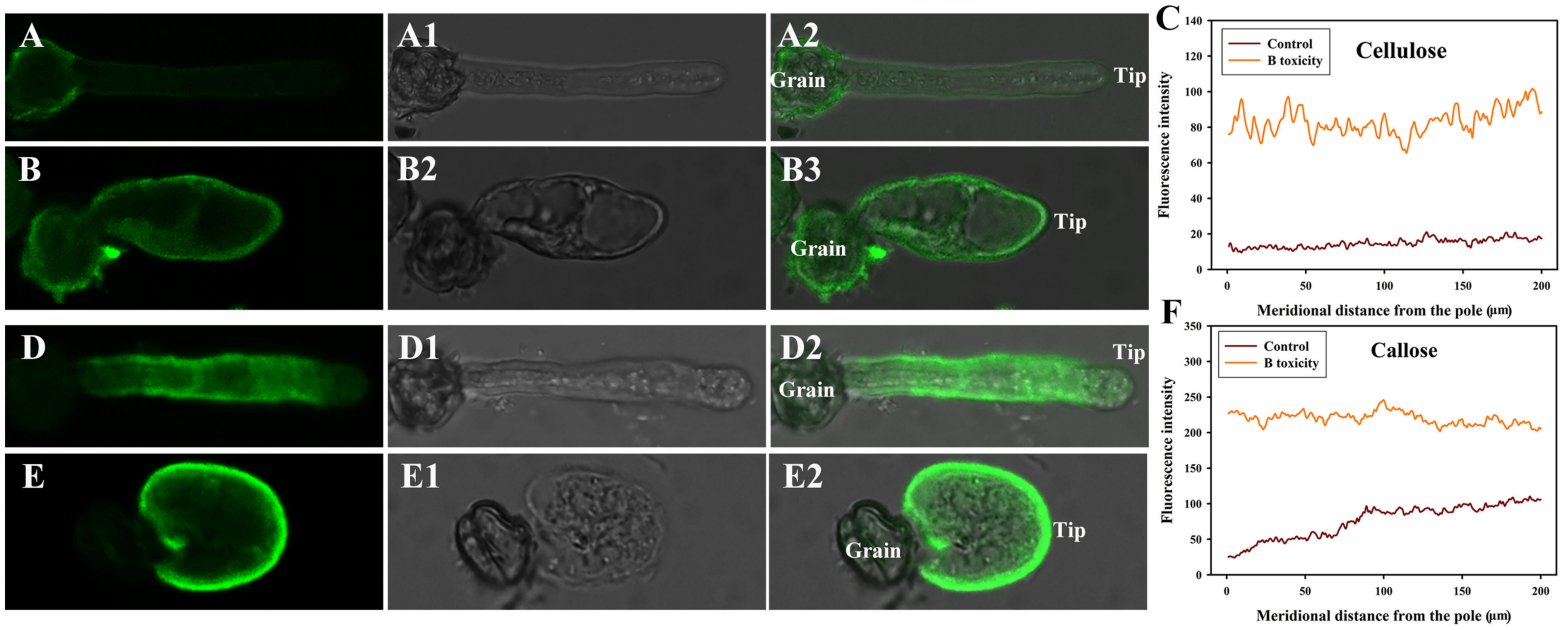
Effects of B on cellulose and callose distribution on *C*. *mollissima* pollen tube wall. **(A)** Cellulose in control pollen tube indicated by CBM3a labeling. **(B)** Cellulose in B toxicity-treated pollen tube. **(C)** Quantitative analysis of the fluorescent intensity of cellulose in the wall of control and B toxicity-treated pollen tubes. **(D)** Callose in control pollen tube indicated by monoclonal antibody to (1 → 3)-β-glucan labeling. **(E)** Callose in B toxicity-treated pollen tube. **(F)** Quantitative analysis of callose fluorescent intensity in the wall of control and B toxicity-treated pollen tubes. Bar = 10 μm.

B toxicity stimulated accumulation of cellulose at the pollen tube tip. Fluorescent signals of cellulose appeared through the control pollen tube except for the tip (Figure 3A). However, B toxicity induced the accumulation of cellulose at the pollen tube tip (Figure 3B). Fluorescent intensity also supported the above results (Figure 3C).

Similar to cellulose distribution, B toxicity stimulated the accumulation of callose at the pollen tube tip. Stronger fluorescent signals appeared through the control pollen tube except for the tip (Figure 3D). However, callose accumulated at the pollen tube tip after B toxicity treatment (Figure 3E). Fluorescent intensity also supported the above results (Figure 3F).

### Effects of B toxicity on pollen tube wall ultrastructure

**Figure 4:**
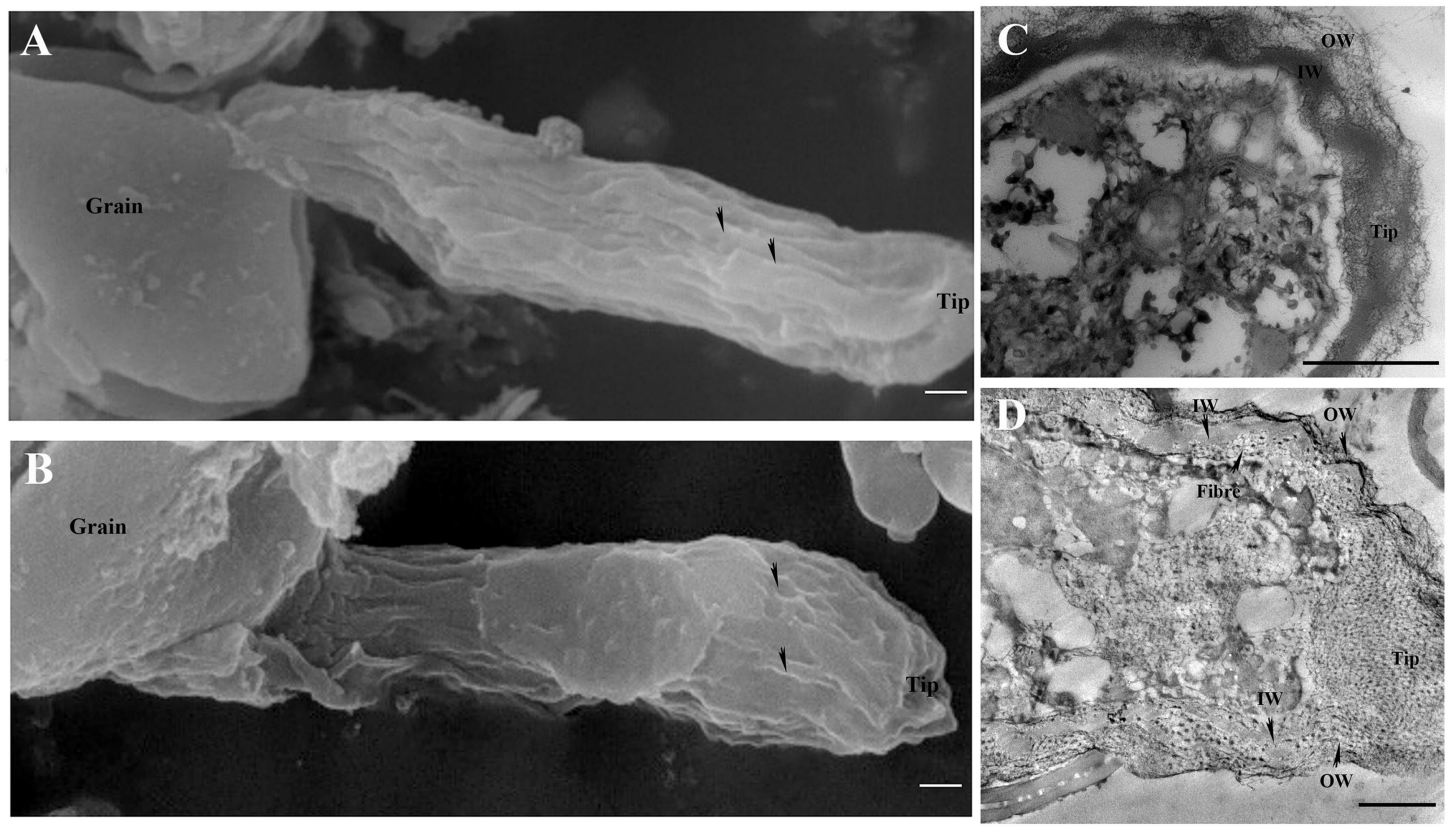
Effects of B toxicity on the ultrastructure of *C*. *mollissima* pollen tubes. **(A)** Scanning electron micrograph (SEM) of the control pollen tube. **(B)** SEM of B toxicity-treated pollen tube. **(C)** TEM transverse section of the control pollen tube. One layer (fibrous wall) exited in the tips, but two layers (fibrous pectic layer in the OW and callose layer in the IW) exited in the tubes. **(D)** TEM transverse section of B toxicity-treated pollen tube. The disorganized fibrous wall existed at the pollen tube tip. And the OW and IW were crosslinked and fiber exited inside of IW. OW, Outer wall; IW, inner wall. Bar = 3 μm in A and B; Bar = 1 μm in **(C,D)**. The arrows indicated longitudinal furrows in A and B.

The surface of the control pollen tube did not appear smooth but instead showed that cell wall material oriented slightly parallel to the direction of the tube elongation (Figure 4A). Control pollen tube was straight with many longitudinal furrows on the surface (Undulating outer layer). There were many longitudinal furrows on the swollen surface of B toxicity-treated pollen tubes (Figure 4B).

There were two layers of walls behind the tip of the control pollen tube namely the outer wall (OW, the fibrous pectic layer) and the inner wall (IW, callose layer) (Figure 4C). Only one layer of wall existed on the tip of the control pollen tube (Figure 4C). However, B toxicity induced two layers of the wall at the pollen tube tip with distorted microfibril at the tip (Figure 4D). And the wall was thicker than that of the control. B toxicity induced deposition of callose at the tip of the pollen tube, resulting in two layers of walls there. In the control pollen tube, the outer wall was thinner at the sub-tip, while it was thicker at the shank. B toxicity led to a very thick and disorganized fibrous outer wall at the tip.

### FTIR spectroscopy analysis of pollen tube wall components

**Figure 5:**
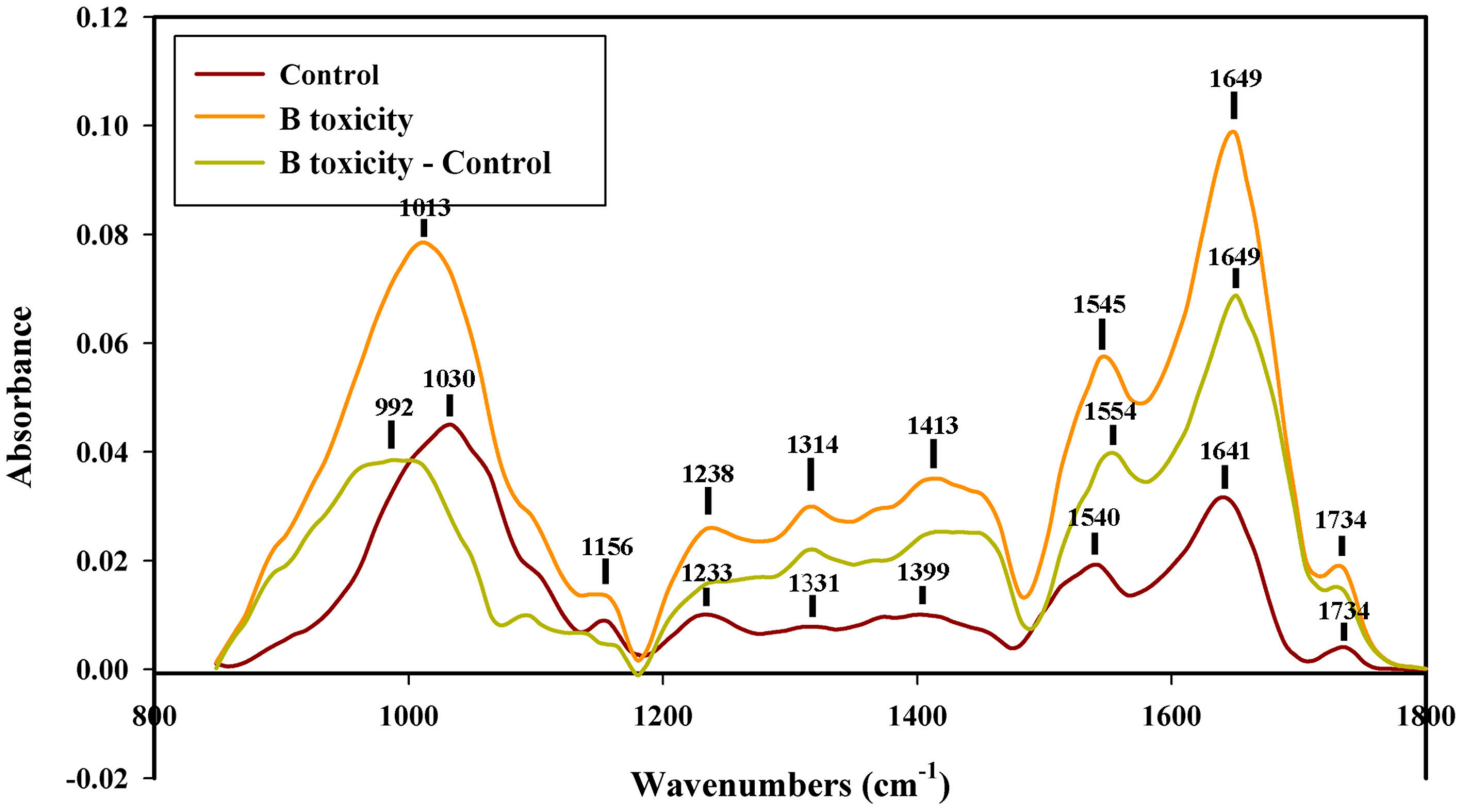
FTIR spectra from the tip regions of *C*. *mollissima* pollen tubes (control pollen tube: Control), B toxicity-treated pollen tubes and the FTIR differential spectrum (B toxicity**–**control) generated by digital subtraction of the control spectra from B toxicity-treated pollen tube spectra.

In the control pollen tube tip spectrum, the distinct peaks of saturated esters were at 1,734 cm^1^, the peaks of protein were at 1,641 and 1,540 cm^−1^, peaks of lignin and carboxylic acid were at 1,641, 1,399, and 1,233 cm^−1^, the peaks of cellulose and hemicellulose were around 1,331 and 1,156 cm^−1^, and the peaks of other polysaccharides were at 1,200 to 900 cm^−1^. However, the peaks of lignin and carboxylic acid, cellulose, pectins, and polysaccharides increased in spectra of pollen tubes treated with B toxicity (Figure 5), indicating that B toxicity influenced the deposition of cell wall components, such as carboxylic acid, pectins, and cellulose on pollen tube cell walls of *C*. *mollissima*.

To study the role of B toxicity on the relationship between pollen tube wall components, cellulase, pectinase, and β-1,3-glucanase were used to treat the pollen tube, respectively. Results are shown below.

### Effects of B toxicity on cell wall architecture with cellulase treatment

**Figure 6:**
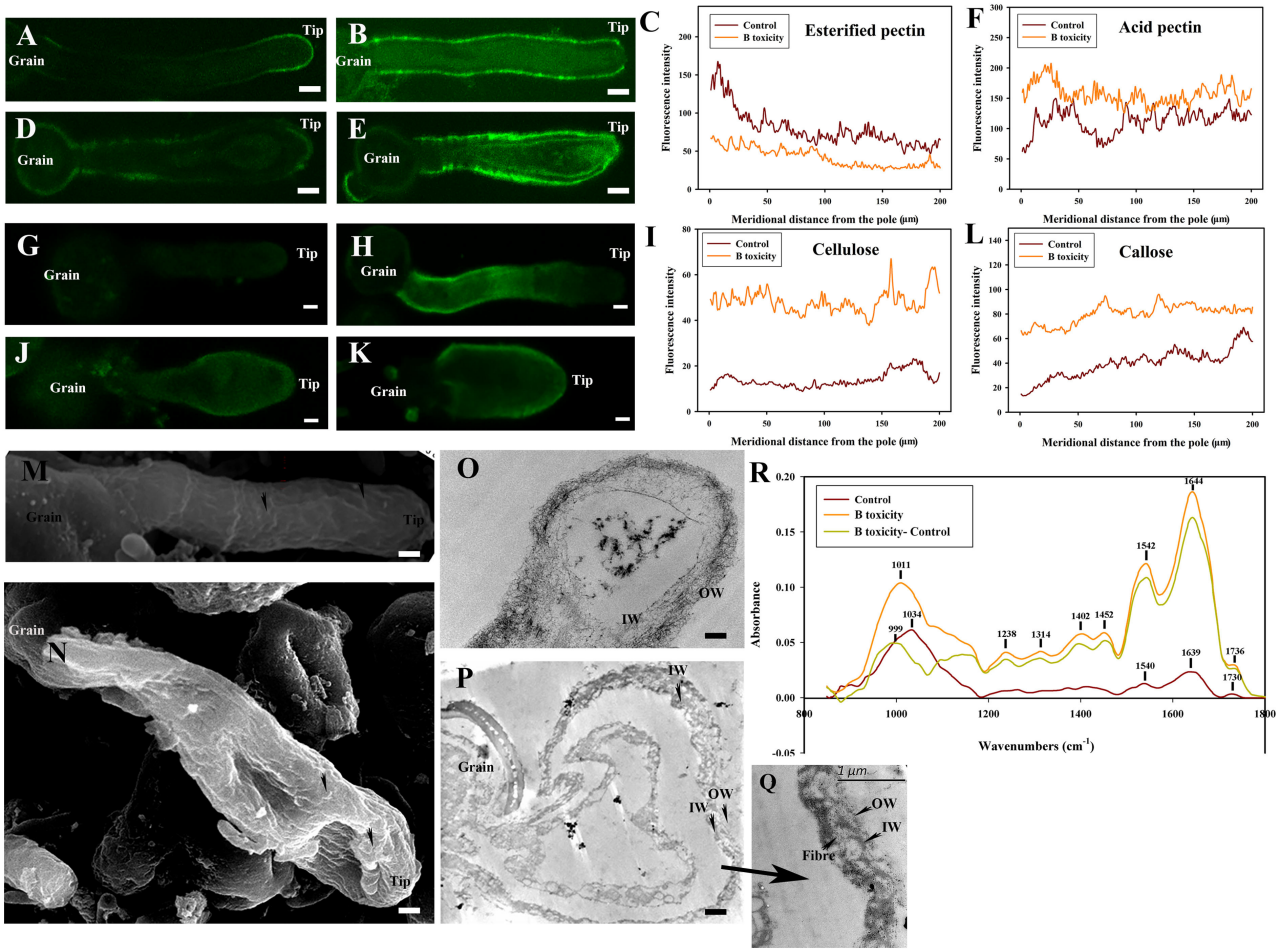
Effects of B toxicity on cell wall architecture with cellulase treatment. **(A)** Esterified pectins labeled with JIM7 in control pollen tube treated with cellulase. **(B)** Esterified pectins in B toxicity-treated pollen tube treated with cellulase. **(C)** Quantitative analysis of the fluorescent intensity of esterified pectins in the wall of control (Control) and B toxicity treated pollen tubes which were digested with cellulase. **(D)** Acid pectins indicated by JIM 5 labeling in control pollen tube treated with cellulase. **(E)** Acid pectins in B toxicity-treated pollen tube treated with cellulase. **(F)** Quantitative analysis of acid pectins fluorescent intensity in the wall of control pollen tubes (Control) and B toxicity-treated pollen tubes which were digested with cellulase. **(G)** Cellulose in control pollen tube digested with cellulase indicated by CBM3a labeling. **(H)** Cellulose in B toxicity-treated pollen tube and then digested with cellulase. **(I)** Quantitative analysis of the fluorescent intensity of cellulose in the wall of control (Control) and B toxicity-treated pollen tubes that were digested with cellulase. **(J)** Callose in control pollen tube indicated by monoclonal antibody to (1 → 3)-β-glucan labeling digested with cellulase. **(K)** Callose in B toxicity-treated pollen tube digested with cellulase. **(L)** Quantitative analysis of callose fluorescent intensity in the wall of control (Control) and B toxicity-treated pollen tubes that were digested with cellulase. **(M)** SEM of control pollen tube digested with cellulase. **(N)** SEM of B toxicity-treated pollen tube digested with cellulase. **(O)** TEM transverse section of control pollen tube digested with cellulase. The fibrous pectic layer in OW became loosened. **(P,Q)** TEM transverse section of B toxicity-treated pollen tube digested with cellulase. A small number of fibers were present in OW, but much more fibers were crosslinked with the callose layer. **(R)** FTIR spectra from the tip regions of *C*. *mollissima* pollen tubes digested with cellulase (control pollen tube: Control), B toxicity-treated pollen tubes digested with cellulase and the FTIR differential spectrum (B toxicity–control) generated by digital subtraction of the control spectra from B toxicity-treated spectra digested with cellulase (B toxicity). Bar = 10 μm in **(A,B,D,E,G,H,J,K)**. Bar = 3 μm in M and N; Bar = 2 μm in **(O,P)**. OW, Outer wall; IW, inner wall. The arrows showed the wave morphology **(M)** and wave protuberances **(N)**.

In treatment with cellulase, the pectins decreased to some extent, obviously esterified pectins in control pollen tubes (Figures 6A–F), and B toxicity reduced signals strongly at the tip. However, the acid pectin signals were still strong after cellulase treatment, and the signals of pollen tubes with B toxicity treatment were stronger than that of control pollen. What's more, the esterified and acid pectins were discontinuously distributed in the tube wall with cellulase treatment.

After cellulase treatment, the cellulose signals of control pollen tubes were detected difficultly, and the cellulose signals of pollen tubes with B toxicity treatment were stronger. But after cellulase treatment, the callose signals intensity had a little decrease in the tube wall, and the signals of pollen tubes with B toxicity treatment were stronger than that of control pollen tubes (Figures 6G–L).

Treatment with cellulase induced more obvious wave morphology at the control pollen tube surface (Figure 6M). There were many wave protuberances on the tip and swollen place of the pollen tube treated by B toxicity. Striped collaterals were distributed on the place except for the tip (Figure 6N).

Treatment with cellulase led to a very thin and loosened outer wall and inner wall of control pollen tubes (Figure 6O). However, it was difficult to distinguish the two layers of the wall under B toxicity treatment; a small number of fibers were present in OW, but much more fibers were crosslinked with the IW (Figures 6P,Q).

Effect of B toxicity on chemical components of pollen tube wall treated with cellulase was carried out with FTIR (Figure 6R). The results showed that the distinct peaks assigned to saturated esters (the peak at 1,734 cm^−1^), proteins (the peaks at 1,639 and 1,540 cm^−1^), lignin and carboxylic acid (the peaks at 1,399, and 1,233 cm^−1^), cellulose and hemicellulose (the peaks around 1,331 and 1,156 cm^−1^), and other polysaccharides (the peaks at 1,200–900 cm^−1^) decreased in the control pollen tube spectrum and B toxicity increased the peaks assigned to lignin and carboxylic acid, cellulose, pectins, and polysaccharides, indicating that B toxicity influenced the deposition of cell wall components, such as carboxylic acid, pectins, and cellulose, in pollen tube cell walls of *C*. *mollissima*.

In a word, treatment with cellulase led to a discontinuous distribution of esterified pectins and acid pectins and decreased cellulose and callose content. There existed an intimate relationship among pectins, cellulose, and callose, which was supported by the ultrastructure.

### Effects of B toxicity on cell wall architecture with pectinase treatment

No fluorescent signals of esterified pectins and acid pectins were detected on control and B toxicity treated pollen tube wall digested with pectinase treatment.

**Figure 7:**
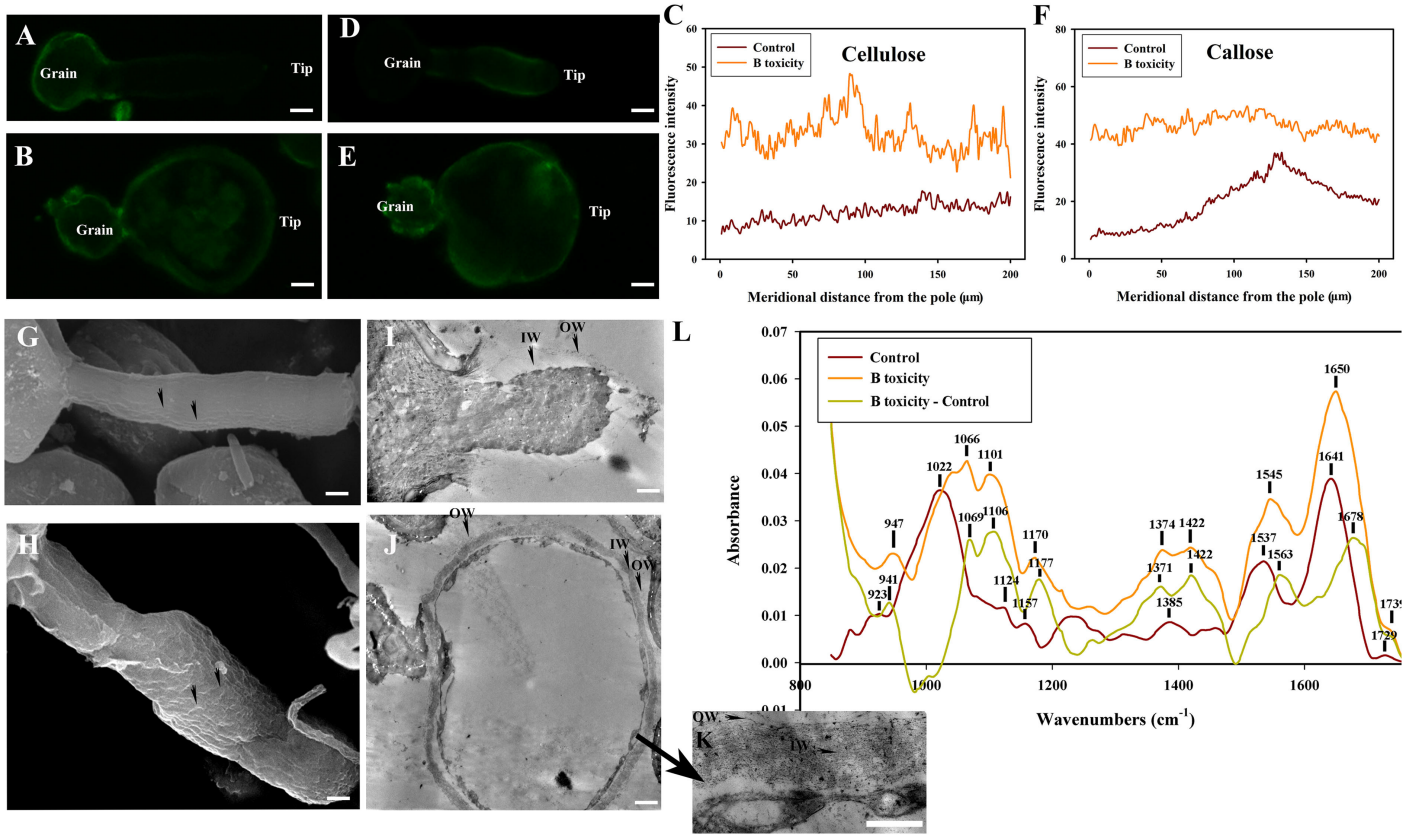
Effects of B toxicity on cell wall architecture with pectinase treatment. **(A)** Cellulose in control pollen tube digested with pectinase indicated by CBM3a labeling. **(B)** Cellulose in B toxicity-treated pollen tube digested with pectinase. **(C)** Quantitative analysis of the fluorescent intensity of cellulose in the wall of control (Control) and B toxicity-treated pollen tubes which were digested with pectinase. **(D)** Callose in control pollen tube indicated by monoclonal antibody to (1 → 3)-β-glucan labeling digested with pectinase. **(E)** Callose in B toxicity-treated pollen tube treated with pectinase. **(F)** Quantitative analysis of callose fluorescent intensity in the wall of control (Control) and B toxicity-treated pollen tubes which were digested with pectinase. **(G)** SEM of control pollen tube digested with pectinase. **(H)** SEM of B toxicity-treated pollen tube digested with pectinase. **(I)** TEM transverse section of control pollen tube digested with pectinase. After pectinase treatment, the fibrous pectic layer reduced substantially, only a small number of fibres exited in OW, and the callose layer was difficult to be observed. **(J,K)** TEM transverse section of B toxicity-treated pollen tube digested with pectinase. A small number of fibers were observed in OW, but the IW of B toxicity-treated pollen tubes was thick, and much more fibers were crosslinked with the callose layer. **(L)** FTIR spectra from the tip regions of *C*. *mollissima* pollen tubes digested with pectinase (control pollen tube: Control), B toxicity-treated pollen tubes digested with pectinase and the FTIR differential spectrum (B toxicity–control) generated by digital subtraction of the control spectra from B toxicity-treated spectra digested with pectinase. Bar = 10 μm in **(A,B,D,E)**. Bar = 3 μm in **(G,H)**; Bar = 2 μm in **(I,J)**. Bar = 1 μm in **(K)**. OW, Outer wall; IW, inner wall. The arrows indicated the strip protuberances **(G)** and wave protuberances **(H)**.

Weaker fluorescent signals of cellulose were detected on the whole control and B toxicity treated pollen tubes, the cellulose signals of B toxicity treated pollen tubes were stronger than in control pollen tubes (Figures 7A–C). Fluorescent signals of callose decreased after pectinase treatment (Figure 7D), but the callose signals at the tips of B toxicity-treated pollen tubes were stronger than that in control pollen tubes (Figures 7E,F).

Treatment with pectinase induced strip protuberances on the control pollen tube surface (Figure 7G). There were many wave protuberances on swollen pollen tubes treated with B toxicity (Figure 7H).

The microfibril of the control pollen tube treated with pectinase reduced substantially, only a small amount of fibers exited in OW and IW was difficult to be observed (Figure 7I). However, a small number of fibers were observed in OW, but the IW of B toxicity-treated pollen tubes was thick and much more fibers were crosslinked with the callose layer (Figures 7J,K).

Effect of B toxicity on chemical components of pollen tube wall treated with pectinase were carried out with FTIR (Figure 7L). The results showed that the distinct peaks of saturated esters (the peak at 1,729 cm^−1^), proteins (the peaks at 1,641 and 1,537 cm^−1^), lignin and carboxylic acid (the peaks at 1,385 and 1,233 cm^−1^), cellulose and hemicellulose (the peaks around 1,331 and 1,157 cm^−1^), and other polysaccharides (the peaks at 1,200–900 cm^−1^) decreased in the control pollen tube spectrum. B toxicity increased the peaks of lignin and carboxylic acid, cellulose, pectins, and polysaccharides and decreased other polysaccharides, indicating that B toxicity influenced the deposition of cell wall components, such as carboxylic acid, pectins, and cellulose, into pollen tube cell walls of *C*. *mollissima*.

In a word, treatment with pectinase resulted in the disappearance of esterified and acid pectins and decreased cellulose and callose. B toxicity caused disordered ultrastructure.

### Effects of B Toxicity on cell wall architecture with β-1,3-glucanase treatment

No fluorescent signals of esterified pectins and acid pectins were detected on control and B toxicity-treated pollen tube wall digested with β-1,3-glucanase indicating that degradation of callose-induced disappearance of pectins.

**Figure 8:**
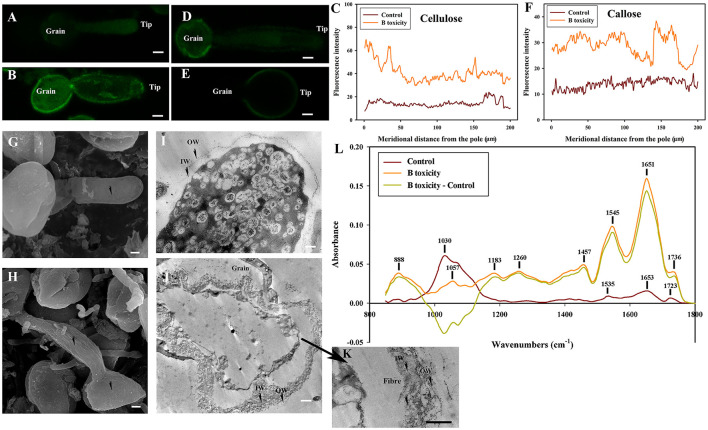
Effects of B toxicity on cell wall architecture treated with β-1,3-glucanase treatment.**(A)** Cellulose in control pollen tube digested with β-1,3-glucanase indicated by CBM3a labeling. **(B)** Cellulose in B toxicity-treated pollen tube digested with β-1,3-glucanase. **(C)** Quantitative analysis of the fluorescent intensity of cellulose in the wall of control (Control) and B toxicity-treated pollen tubes, which were digested with β-1,3-glucanase. **(D)** Callose in control pollen tube indicated by monoclonal antibody to (1 → 3)-β-glucan labeling digested with β-1,3-glucanase. **(E)** Callose in B toxicity-treated pollen tube digested with β-1,3-glucanase. **(F)** Quantitative analysis of callose fluorescent intensity in the wall of control (Control) and B toxicity-treated pollen tubes which were digested with β-1,3-glucanase. **(G)** SEM of control pollen tube digested with β-1,3-glucanase. **(H)** SEM of B toxicity-treated pollen tube digested with β-1,3-glucanase. **(I)** TEM transverse section of control pollen tube digested with β-1,3-glucanase. A thin fiber pectin layer in OW of control pollen tubes was observed and the IW was difficult to be observed with β-1,3-glucanase. **(J,K)** TEM transverse section of pollen tube treated with β-1,3-glucanase. After β-1,3-glucanase treatment, the fiber pectin layer in OW was observed with a thin layer, but the IW of B toxicity-treated pollen tubes was still thick, and there were still some fibers crosslinked with the callose layer to form an irregular crosslinking layer. **(L)** FTIR spectra from the tip regions of *C*. *mollissima* pollen tubes digested with β-1,3-glucanase (control pollen tube: Control), B toxicity-treated pollen tubes treated with β-1,3-glucanase and the FTIR differential spectrum (B toxicity–control) generated by digital subtraction of the control spectra from B toxicity-treated spectra digested with β-1,3-glucanase. Bar = 10 μm in **(A,B,D,E)**. Bar = 3 μm in G and H; Bar = 2 μm in **(I,J)**. Bar=1 μm in K. OW, Outer wall; IW, inner wall. The arrows showed the smooth surface **(G)** and the uneven surface **(H)**.

Weaker fluorescent signals of cellulose were detected on the whole control pollen tube digested with β-1,3-glucanase (Figure 8A), but fluorescent signals of cellulose were stronger on B toxicity-treated pollen tubes digested with β-1,3-glucanase, especially at the tips (Figures 8B,C). After β-1,3-glucanase treatment, fluorescent signals of callose decreased, and appeared through the control pollen tube except for the tip (Figure 8D). However, B toxicity induced accumulation of callose at the pollen tube shank and tip (Figures 8E,F).

The surface was very smooth in control pollen tubes treated with β-1,3-glucanase (Figure 8G), while B toxicity led to an uneven surface of pollen tubs with swollen tips (Figure 8H).

Very thin outer wall were observed in the control pollen tubes, and the IW was difficult to be observed with β-1,3-glucanase (Figure 8I). The fibrous pectic layer reduced substantially and only a thin layer exited in OW of B toxicity-treated pollen tubes after β-1,3-glucanase treatment, but the IW of B toxicity-treated pollen tubes was still thick, and there were still some fibers crosslinked with the callose in IW to form an irregular crosslinking layer (Figures 8J,K).

Effect of B toxicity on chemical components of pollen tube wall treated with β-1,3-glucanase were carried out with FTIR (Figure 8L). The results showed that the peaks assigned to saturated esters (the peak at 1,723 cm^−1^), proteins (the peaks at 1,653 and 1.535 cm^−1^), carboxylic acid (1,457 cm^−1^), and carbohydrates (the peaks at 1,200–900 cm^−1^) decreased in the control pollen tube spectrum treated with β-1,3-glucanase. However, B toxicity increased the peaks assigned to esters and carboxylic acid and decreased carbohydrates, indicating that B toxicity influenced the deposition of cell wall components, such as carboxylic acid, pectins, and cellulose, into pollen tube cell walls of *C*. *mollissima*.

In a word, treatment with β-1,3-glucanase induced the disappearance of esterified and acid pectins and decreased cellulose and callose of control pollen tubes. B toxicity increased cellulose and callose on the pollen tubes. The degradation of callose decreased cellulose and pectins. The result illustrated that there existed an intimate relationship among cellulose, callose, and pectins, which was supported by the ultrastructure.

## Discussion

Although B is an essential micronutrient for plant growth, an excessive concentration of B due to arid and saline soils, as well as low rainfall and poor irrigation, usually produces toxicity in plants (Matthes et al., 2020). Many research illustrated that B toxicity affected mechanical properties of the plant cell wall and thus affected various developmental processes in plants (Landi et al., 2019). In one model of tip growth–root hair, the inhibition of root elongation has been determined to be one of the most distinct symptoms induced by B toxicity in plants (Tanaka and Fujiwara, 2008; Aquea et al., 2012). In another model of tip growth–pollen tube, we found that B toxicity inhibited apple pollen tube elongation through [Ca^2+^] signals and altered tube wall components (Fang et al., 2016). In the present study, we found the growth of pollen tubes were not affected by B toxicity significantly, but the tips of tubes bulged sharply, which was different from our previous studies of apple pollen tubes, and B toxicity induced smaller bulge in apple pollen tubes (Fang et al., 2016). In the study of *Arabidopsis* pollen tube, the over-expression of ANXUR receptor-like kinases (RLKs) inhibited growth and increased the width of pollen tubes by over-activating exocytosis and the over-accumulation of secreted wall materials (Boisson-Dernier et al., 2013). We speculated that B toxicity induced an increase in exocytosis and accumulated deposition of cell wall material to result in the bulge of the pollen tube tip. Also, in this study, we focused on changes in the pollen tube wall components induced by B toxicity with emphasis on ultrastructure. Our present work provides novel evidence for the effects of B toxicity on pollen tube wall components and ultrastructure.

As we mentioned above, B toxicity resulted in less agricultural production. While pollen tube development is critical for double fertilization, we speculate that the disorder of pollen tubes leads to less fruit production. Huang et al. (2016) reported that B toxicity led to the secondary deposition of cell-wall components in regions close to the pits of vessel elements in tolerant *Castanea sinensis* and poorly developed vessel elements in intolerant *Castanea grandis*. In the present research, B toxicity led to fibrous wall thickening at the tube tip and therefore swollen tip.

### Effects of B toxicity on the deposition of cell wall components

As we mentioned above, the pollen tube wall consists of cellulose, callose, and pectins (Li et al., 1996, 2002; Ferguson et al., 1998). The interactions between pectins and B have been reported to be important for the assembly of the cell wall (Baluška et al., 2003). Two rhamnogalacturonan II (RG-II) chains of pectins could be linked with B by forming borate-diol ester bonds (Funakawa and Miwa, 2015), which is essential to cell wall formation (Dumont et al., 2014). The Golgi apparatus produces and transports esterified pectins to the pollen tube extreme tip (Hasegawa et al., 1998), where the enzyme pectin methyl-esterase could alter the esterified pectins to acid pectins (Li et al., 2002). The crosslinks between the acidic residue of acid pectins and Ca^2+^ ions form a semi-rigid pectate gel (Braccini and Perez, 2001). So the changes in B concentrations may cause a mechanical change in the cell wall. B toxicity has been reported to alter cell-wall polysaccharides as well as other cell-wall components in *Citrus* leaf veins and roots (Wu et al., 2019). In our previous research, B toxicity also induced changes in apple pollen tube wall components including acid pectins and esterified pectins (Fang et al., 2016). In the present research, cellulose was present throughout the control pollen tube wall except for the tip. However, B toxicity induced cellulose deposition at the pollen tube tip. Callose is one of the important components in the normal pollen tube walls (Li et al., 2002). In addition, callose is also found at the abnormal pollen tube tip (Hao et al., 2013). Our previous results demonstrated that B toxicity induced the deposition of the callose at the pollen tube tip of *Malus domestica* (Fang et al., 2016). In the present study, similar results were obtained. In the control pollen tube, callose was distributed along the entire pollen tube except for the tip, while B toxicity induced accumulation of callose at the pollen tube tip.

### Effects of B toxicity on the relationship among pectins, cellulose, and callose

In apple pollen tubes, B toxicity had no obvious effect on cellulose distribution. On the contrary, B toxicity enhanced callose deposition at the pollen tube tip and increased acid pectins and esterified pectins at the pollen tube (Fang et al., 2016). In the present study, similar results were obtained. B toxicity induced accumulation of the cellulose and callose at the tip and increased esterified and acid pectins.

In the present study, to further study the effects of B toxicity on the relationship among pectins, cellulose, and callose, cellulase, pectinase, and β-1,3-glucanase were applied. Results from FTIR showed that treatment with cellulase, pectinase, and β-1,3-glucanase decreased chemical components of the pollen tube wall, suggesting the intimate relationship among cellulose, pectins, and callose. β-1,3-glucanase induced the disappearance of the pectins in pollen tubes, indicating that there existed closer relation between callose and pectins. Also, the pectin signals after cellulase treatment showed intermittent distribution, and disappeared after β-1,3-glucanase treatment, which suggested the crossliking relationship of cellulose, pectins, and callose of tube walls. And we speculated that the relationship between pectins and callose might be stronger than that between cellulose and pectins. However, B toxicity not only affected the morphology of the pollen tube but also affected the tube wall components. In addition, B toxicity strengthened the relationship among the pectins, cellulose, and callose with stronger fluorescence and higher peak of chemical components of the pollen tube wall.

### Effect of B toxicity on pollen tube cell wall ultrastructure

B toxicity altered the cell wall structure. B toxicity thickened the cell wall in cork cells of *C. grandis* leaves (Huang et al., 2014). B toxicity has been reported to alter the pectin network crosslinking structure of leaf tip and cell ultrastructure and architecture of components in leaf center and the tip of trifoliate orange (Wu et al., 2019). Although B is essential for pollen tube wall morphological formation, few investigations reported the effect of B toxicity on pollen tube wall ultrastructure. In our present study, B toxicity resulted in the thicker wall at the pollen tube tip and destruction of cell wall integrity leading to the swollen tip.

In brief, we found that boron toxicity disturbed cell wall components such as pectins, cellulose, callose, and ultrastructure. B toxicity also altered the relation among pectins, cellulose, and callose. Our investigation of the effects of boron toxicity on *C. mollissima* pollen tubes provides an extensive understanding of the role of boron in the cell wall component architecture of pollen tubes. These findings provide valuable information which enhances our current understanding of the mechanism regulating B in pollen tube growth.

## Data availability statement

The original contributions presented in the study are included in the article/Supplementary material, further inquiries can be directed to the corresponding authors.

## Author contributions

KF and LQ designed the research and edited the article. FK drafted the article. WZ performed the main experiments. QZ, QC, and YX were involved in data analysis. All authors have read and approved the final version of the manuscript.

## Funding

This work was supported by the National Key Research and Development Program of China (2018YFD1000605), Beijing advanced innovation center for tree breeding by molecular design (2023210001) and the National Natural Science Foundation of China (31270719).

## Conflict of interest

Author WZ was employed by Beijing Bei Nong Enterprise Management Co. Ltd. The remaining authors declare that the research was conducted in the absence of any commercial or financial relationships that could be construed as a potential conflict of interest.

## Publisher's note

All claims expressed in this article are solely those of the authors and do not necessarily represent those of their affiliated organizations, or those of the publisher, the editors and the reviewers. Any product that may be evaluated in this article, or claim that may be made by its manufacturer, is not guaranteed or endorsed by the publisher.
