# Supplementary material for: Effect of boron toxicity on pollen tube cell wall architecture and the relationship of cell wall components of Castanea mollissima Blume
Source: Front Plant Sci. 2022 Jul 26;13:946781. doi: 10.3389/fpls.2022.946781 (PMC9361862; doi:10.3389/fpls.2022.946781)
Supplement: Supplementary file 1 [file Table_1.DOCX]

Table 1 **Germination rate and length of** *C*. *mollissima* **pollen tubes under different concentrations of H_3_BO_3_**

| H_3_BO_3_ treatment/ mM | Germination rate/% | Pollen tube length/µm | Pollen tube width/µm |
| --- | --- | --- | --- |
| 0 | 21.89±1.68 ab | 25.80±0.75 b | 5.53±0.26 c |
| 0.08 | 31.60 ±0.45 a | 38.22±0.36 a | 5.71±0.20 c |
| 0.4 | 19.78±1.55 b | 39.10±0.53 a | 16.23±3.34 b |
| 0.8 | 14.57±1.04 c | 39.82±2.84 a | 20.91±1.37 a |

Note: Different letters indicate statistically significant differences between pollen tubes grown in various condition (*P*≤0.05).
